# Association between thyroid function and prognosis of severe COVID-19 among patients with SARS-CoV-2 infection: a retrospective cohort study in China

**DOI:** 10.3389/fendo.2024.1361479

**Published:** 2024-09-24

**Authors:** Yaling Yang, Lifang Qian, Chenwei Wu, Xinyue Xu, Duoduo Qu, Lihua Zhou, Jia Liu, Qin Zhu, Chunhong Wang, Xiaolong Zhao

**Affiliations:** ^1^ Department of Endocrinology, Shanghai Public Health Clinical Center, Shanghai, China; ^2^ Department of Medicine, Shanghai Qingpu District Zhujiajiao People’s Hospital, Shanghai, China

**Keywords:** SARS-CoV-2, severe COVID-19, non-thyroid illness syndrome, thyroid function, total triiodothyronine

## Abstract

**Objective:**

This study aims to examine the thyroid hormone profile and its association with severe coronavirus disease 2019 (COVID-19) in patients infected by severe acute respiratory syndrome coronavirus 2 (SARS-CoV-2).

**Methods:**

This retrospective cohort study enrolled patients admitted to a tertiary hospital due to SARS-CoV-2 infection between February 18 and May 18, 2022. Clinical data were collected retrospectively from the electronic medical record system. Based on the thyroid function, patients were divided into five groups: normal, non-thyroid illness syndrome (NTIS), hypothyroidism, thyrotoxicosis, and unclassified. The association between thyroid function and severe COVID-19 was detected using multivariable logistic regression and restricted cubic splines analysis.

**Results:**

This study included 3,161 patients, with 7.7% of them developing severe COVID-19. 44.9% of the patients had normal thyroid function, 36.5% had NTIS, 6.7% had hypothyroidism, and 1.0% had thyrotoxicosis on admission. After adjusting for age, sex, and relevant clinical characteristics, NTIS and hypothyroidism were associated with increased risks of severe COVID-19 (odds ratio [OR] 2.38, 95% confidence interval [CI] 1.59-3.56 and OR 2.29, 95% CI 1.23-4.26, respectively), compared to normal thyroid function group. Among patients with NTIS or hypothyroidism, higher levels of total triiodothyronine (TT3) are associated with lower risks of severe COVID-19 (OR 0.73, 95% CI 0.64-0.82, for every 0.1nmol/L increase in TT3 level).

**Conclusion:**

Thyroid hormone profiles of NTIS or hypothyroidism are associated with increased risks of severe COVID-19. The decreased level of TT3 correlated with the increased risk of severe COVID-19 in patients with NTIS or hypothyroidism.

## Introduction

Since it first emerged in 2019, the Coronavirus disease-2019 (COVID-19) pandemic has had a profound and far-reaching impact on global health. It continues to pose a significant and ongoing threat to public well-being. This disease presents a wide array of symptoms, ranging from mild to severe and even fatal, affecting multiple organs and systems of the human body, including the endocrine glands and the thyroid gland (1).

Following the publication of a case report on subacute thyroiditis after infection with severe acute respiratory syndrome coronavirus 2 (SARS-CoV-2) by Brancatella et al. (2), considerable attention has been given to the research on the relationship between COVID-19 and the thyroid gland (3). However, as more clinical evidence accumulated, a wide range of thyroid function abnormalities have been noticed, including thyroiditis, non-thyroidal illness syndrome (NTIS), clinical and subclinical hypothyroidism, central hypothyroidism, and clinical and subclinical hyperthyroidism (4). It is now well recognized that NTIS, characterized by low levels of triiodothyronine (T3) and normal or low levels of thyroid-stimulating hormone (TSH) (5, 6), is quite common among patients with SARS-CoV-2 infection (5, 7–10).

NTIS has been reported to be a complex condition associated with severe diseases, such as poor nutrition, heart failure, chronic obstructive pulmonary (COPD), and community-acquired pneumonia (6, 11). Some scholars have concluded that NTIS could predict clinical deterioration in COVID-19 patients (9, 12). And some studies have found that low triiodothyronine levels hold significant predictive value for COVID-19 patients (13, 14). These results have motivated us to conduct further research. In this study, we conducted a retrospective analysis to investigate the thyroid hormone profiles, especially NTIS, in SARS-CoV-2 infected patients. We also explored the relationship between triiodothyronine and the risk of developing severe COVID-19 during hospital stays and examined the predictive value of thyroid function tested on admission.

## Methods

### Study population

This retrospective cohort study analyzed data from inpatients in a tertiary hospital during the 2022 COVID-19 outbreak in Shanghai. The study subjects were adults (≥18 years) admitted due to SARS-CoV-2 infection from February 18 to May 18, 2022. Clinical Data were collected retrospectively from the electronic medical record system. The subjects were all Chinese residents with routine clinical data and laboratory tests, including complete blood count, renal and liver function, thyroid function, and coagulation function, which were conducted on admission.

### Thyroid function

Thyroid function was tested within 24 hours after admission, including total triiodothyronine (TT3), total thyroxine (TT4), free triiodothyronine (FT3), free thyroxine (FT4), and TSH. Based on the thyroid hormone levels, the thyroid hormone patterns were categorized into five groups: NTIS, hypothyroidism, thyrotoxicosis, normal, and unclassified.

1. NTIS was defined as low TT3 with either normal TSH or low TSH (15).
2. Hypothyroidism was defined as overt hypothyroidism (high TSH and low FT4) or subclinical hypothyroidism (high TSH and normal FT4) (16).
3. Thyrotoxicosis was defined as overt thyrotoxicosis (low TSH, high TT3, and high FT4), mild (low TSH, high TT3, and normal FT4), or subclinical thyrotoxicosis (low TSH, normal TT3, and normal FT4) (17).
4. The normal group was defined as normal TSH, TT3, and FT4 levels.
5. The unclassified group includes individuals with thyroid hormone profiles that do not fit the criteria mentioned above. This includes:
  - Normal TSH, TT3, and high or low FT4.
  - Normal TSH, FT4, and high TT3.
  - High TSH, FT4, and low or normal TT3.
  - Low TSH, normal TT3, and high FT4.

### Covariates

Covariates included age, gender, diabetes, vaccination status, and laboratory tests examined within 24 hours of admission, which included complete blood count, renal and liver function, thyroid function, and coagulation function. The combination of diabetes was established based on a prior diabetes diagnosis or an HbA1C level of ≥6.5% on admission. The vaccinated status of individuals was determined based on having received at least one dose of any COVID-19 vaccine.

### Clinical outcomes

The primary outcome is the occurrence of severe COVID-19, which was determined by a particular expert group after discussion, according to Chinese Clinical Guidance for COVID-19 Pneumonia Diagnosis and Treatment (Trial Version 9) (18), when patients met any of the following criteria: respiratory rate ≥30/min, SpO2 ≤93% at rest, and >50% progression in 48 h on imaging, critical disease state which was defined as respiratory failure requiring mechanical ventilation, shock, and intensive care unit (ICU) admission.

Secondary outcomes encompassed several variables: 1. Oxygen therapy status, categorized as no oxygen treatment, oxygen inhalation, non-invasive mechanical ventilation, or invasive mechanical ventilation. 2. Hemodialysis treatment, administered during the hospital stay. 3. Time to SARS-CoV-2 RNA turning negative, defined as the number of days from confirming SARS-CoV-2 infection to when SARS-CoV-2 RNA tests showed cycle threshold (Ct) <35 for at least two consecutive days. 4. Length of hospitalization, defined as the number of days between the date of discharge and the initial date of hospitalization. All the clinical outcome data were retrospectively extracted from the hospital information system’s electronic medical record.

### Laboratory measurement

SARS-CoV-2 infection was defined as SARS-CoV-2 RNA with the Ct value <35 in either target gene at least twice. Samples were extracted from nasal/throat swabs and detected with SARS-CoV-2 real-time polymerase chain reaction (RT-PCR) by the dual-target (ORF1ab and N genes), using detection kits from DAAN Inc.

Thyroid function was measured using automated competitive immunoassays: Alinity I (Abbott) according to the manufacturer’s protocol. The normal range is 0.98-2.33 nmol/L for TT3, 62.68-150.84 nmol/L for TT4, 2.43-6.01 pmol/L for FT3, 9.01-19.05 pmol/L for FT4, and 0.35-4.94 uIU/mL for TSH.

Serum amyloid A (SAA) was performed using a specific protein analyzer and accompanying reagents manufactured by Upper Bio-teach Pharma Co. in Shanghai, China. The methods were Particle-Enhanced immunoturbidimetric. All items were operated strictly with the operating procedures, reagent instructions, and indoor quality control.

High-sensitivity C-reactive protein (hs-CRP) was measured using the hs-CRP Assay Kit produced by Shenzhen Lifotronic Technology Co. through scatter turbidimetry, according to the manufacturer’s protocol.

Interleukin-6 (IL-6) was measured using the cytokine detection kits produced by Qingdao Raisecare Biological Technology Co. using the multiple microsphere flow immunofluorescence luminescence method.

### Statistical analysis

Continuous variables were reported as the median and interquartile range (IQR) since most of the variables were not distributed normally. Category variables were reported as frequency and percentage. Demographic and clinical characteristics of the patients were compared between the normal thyroid function group and other thyroid function groups using the Wilcoxon rank-sum or Kruskal–Wallis H test for continuous variables and the chi-square test or Fisher’s exact test for categorical variables.

Initially, we conducted univariate logistic regression to assess the association between thyroid hormone patterns and severe COVID-19. We adjusted for age, sex, diabetes, and vaccination in Model 1. In Model 2, we additionally adjusted aspartate transaminase (AST) levels exceeding three times the upper normal limit (ULN), estimated glomerular filtration rate (eGFR) below 30 ml/min/1.73m^2^, serum calcium (Ca), white blood cell count (WBC), neutrophil-lymphocyte ratio (N/ly), and D-Dimer. Odds ratios (OR) with corresponding 95% confidence intervals (CI) were reported. Missing data were filled using the K-nearest neighbors (KNN) method with a value of K=10.

To evaluate the association between TT3 levels and severe COVID-19, we used a logistic regression model with restricted cubic splines (RCS) to examine nonlinear relationships among all patients affected with SARS-CoV-2. The logistic regression was adjusted for the same baseline clinical characteristics as in Model 2. The median value of the predictor variable was chosen as the reference value. If the curve exhibited a U-shape, Inverted U-shape, or L-shape, the inflection point (i.e., the point where the curve changed its direction) was set as the cut-off value. The correlations between TT3 and inflammatory factors and lymphocyte subsets, stratified by thyroid function pattern, were determined by the Spearman correlation coefficient.

Statistical analyses were performed using SPSS 26 (SPSS/IBM, Armonk, NY, USA). RCS analyses were performed using R software, version 4.2.2, with the rms package. P value <0.05 was considered statistically significant, and a two-tailed P value was reported.

## Results

### Study participants

This study was conducted from February 18 to May 18, 2022, during the prevalence of the BA.2.2 variant of SARS-CoV-2. A total of 3636 hospitalized patients admitted due to SARS-CoV-2 infection were initially screened. Among this group, sixty-eight patients were excluded due to reinfection with SARS-CoV-2, and an additional four hundred and seven patients were excluded because their thyroid function was not tested. Ultimately, 3161 patients were included in this study.

### Thyroid hormone profile of patients on admission

**Figure 1:**
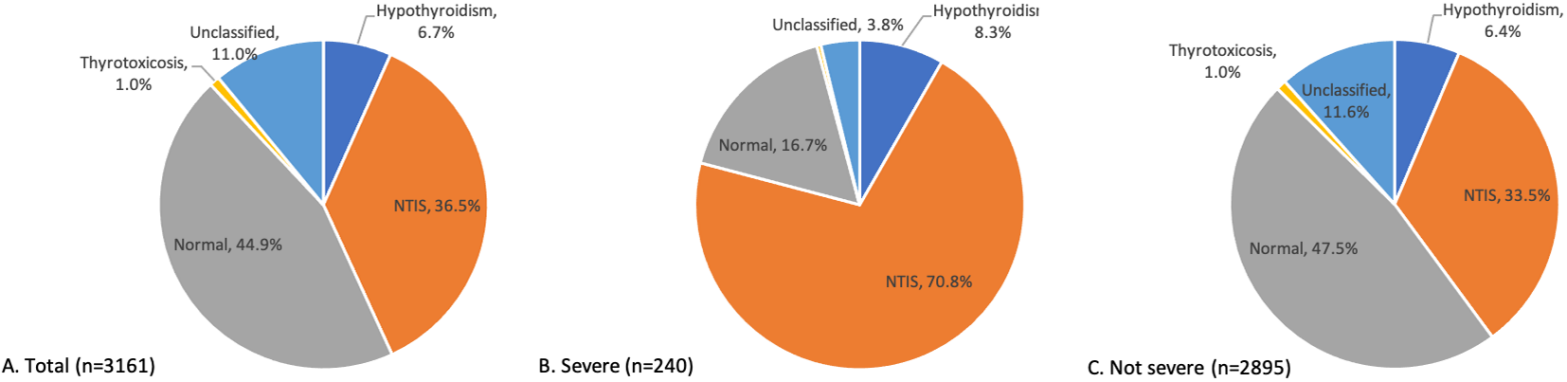
Distribution of thyroid function patterns on admission. Distribution of thyroid function patterns on admission among all the patients **(A)**, severe COVID-19 patients **(B)**, and not severe COVID-19 patients **(C)**.

Normal thyroid dysfunction was present in 44.9% of the 3161 patients included in this study, as shown in **Figure 1A**. NTIS is the most common form of abnormal thyroid dysfunction, affecting 36.5% of the entire study population. Hypothyroidism was found in 6.7% of the patients, with overt hypothyroidism accounted for only 0.16% of the study cohort. Thyrotoxicosis was observed in 1.0% of the study population, while overt thyrotoxicosis was rare, with only two patients (0.06%) exhibiting this condition. The unclassified group comprised 11.0% of the study population. Within the unclassified group, 89% of the patients presented with only high FT4 levels (309 out of 347). 4.6% presented with normal TT3, low TSH, and high FT4, and 3.5% presented with normal TT3, high TSH, and high FT4. For more detailed information, please refer to **Supplementary Table 1**. Among the patients who developed severe COVID-19 during hospitalization, the proportion of nonthyroidal illness syndrome (NTIS) was significantly higher compared to non-severe patients (70.8% vs. 33.5%) on admission (**Figures 1B, C**).

### Clinical characteristics of patients according to thyroid hormone patterns

The clinical characteristics are presented in **Table 1**. The median age of the patients was 60 years (IQR 42, 75); 1657 (52.4%) were male, 28.3% had diabetes, and 54.2% had received at least one dose of the COVID-19 vaccine. Compared to normal thyroid function patients, NTIS patients were much older: median 67 vs 54 years and had more patients with diabetes (36.1% vs 26.3%). In general, NTIS patients had worse conditions in liver and kidney function tests, such as higher lactate dehydrogenase (LDH) levels, lower albumin (ALB), and lower eGFR (median 91 vs. 102, ml/min/1.73m^2^). NTIS patients had lower serum Ca levels (median 1.99 vs. 2.06 mmol/L) and higher D-Dimer levels (median 0.53 vs. 0.26 ug/ml). Hypothyroid patients, when compared to normal thyroid function patients, were older (median 63 vs. 54 years) and less male (40.8% vs. 54.4%). Additionally, hypothyroid patients had higher LDH (median 215 vs. 200, U/L), lower eGFR and Ca levels, and higher D-Dimer levels than the normal thyroid function patients.

**Table 1:** Baseline characteristics and COVID-19 clinical outcome in different thyroid function patterns.

Characteristics	Total	Normal	NTIS	Hypothyroidism	Thyrotoxicosis	Unclassified	Missing, n (%)
Patient, n (%)	3161	1418 (44.9%)	1154 (36.5%)	211 (6.7%)	31 (1.0%)	347 (11.0%)	
Baseline Characteristics							
Age, years	60 (42, 75)	54 (38, 69)	67 (48, 83) *	63 (48, 81) *	40 (32, 69)	62 (43, 74) *	0
Age ≥60 years, n (%)	1595 (50.5%)	585 (41.3%)	699 (60.6%) *	117 (55.5%) *	11 (35.5%)	183 (52.7%) *	0
Male, n (%)	1657 (52.4%)	771 (54.4%)	596 (51.6%)	86 (40.8%) *	15 (48.4%)	189 (54.5%)	0
Diabetes, n (%)	896 (28.3%)	341 (26.3%)	389 (36.1%) *	55 (28.9%)	10 (35.7%)	101 (31.3%)	245 (7.8%)
Vaccinated, n (%)	1714 (54.2%)	884 (66.9%)	496 (46.6%) *	114 (58.8%)	17 (60.7%)	203 (61.3%)	221 (7.0%)
Waiting time^1^, days	1 (1, 6)	2 (1, 8)	1 (1, 4) *	2 (1, 8)	1 (1, 1) *	1 (1, 5) *	30 (0.9%)
Laboratory tests							
HbA1c, %	5.9 (5.6, 6.4)	5.9 (5.5, 6.3)	6.0 (5.6, 6.5) *	5.9 (5.6, 6.4)	5.9 (5.6, 6.6)	6.0 (5.6, 6.5) *	83 (2.6%)
ALT, U/L	18 (12, 28)	19 (13, 31)	17 (11, 26) *	18 (12, 29)	17 (12, 28)	19 (12, 29)	43 (1.4%)
AST, U/L	20 (16.27)	20 (16, 26)	21 (16, 29) *	22 (17, 30)	18 (15, 26)	20 (16, 26)	7 (0.2%)
LDH, U/L	204 (177, 242)	200 (174, 230)	211 (180, 257) *	215 (183, 254) *	195 (161, 227)	202 (178, 242)	9 (0.3%)
ALB, g/L	40 (37, 43)	41 (39, 44)	39 (34, 42) *	40 (36, 43) *	40 (37, 42)	41 (38, 43)	3 (0.1%)
eGFR, ml/min/1.73m^2^	97.4 (79.1, 115.2)	101.5 (85.6, 116.6)	90.8 (67.7, 112.6) *	90.4 (73.0, 112.3) *	106.2 (70.1, 128.6)	98.8 (83.2, 116.0)	6 (0.2%)
AST≥3ULN	55 (1.7%)	9 (0.6%)	42 (3.6%) *	1 (0.5%)	0 (0.0%)	3 (0.9%)	7 (0.2%)
eGFR<30 ml/min/1.73m^2^, n (%)	126 (4.0%)	13 (0.9%)	99 (8.6%) *	12 (5.7%) *	1 (3.3%)	1 (0.3%)	6 (0.2%)
K, mmol/L	3.8 (3.5, 4.1)	3.8 (3.5, 4.0)	3.8 (3.5, 4.1)	3.8 (3.6, 4.1)	3.7 (3.4, 4.0)	3.8 (3.5, 4.1)	8 (0.3%)
Na, mmol/L	39 (138, 141)	140 (138, 141)	139 (137, 141) *	140 (138, 141)	138 (136, 139) *	140 (138, 141)	7 (0.2%)
Ca, mmol/L	2.04 (1.92, 2.18)	2.06 (1.95, 2.19)	1.99 (1.88, 2.15) *	2.02 (1.90, 2.18) *	2.07 (1.94, 2.15)	2.08 (1.95, 2.21)	78 (2.5%)
TT3, nmol/L	1.04 (0.88, 1.22)	1.18 (1.07, 1.35)	0.84 (0.75, 0.91) *	1.06 (0.89, 1.26) *	1.23 (1.08, 1.33)	1.14 (1.05, 1.24) *	3 (0.1%)
TT4, nmol/L	8.2 (7.1, 13.0)	8.7 (7.5, 95.7)	7.4 (6.5, 8.7) *	7.7 (6.6, 55.0) *	10.4 (8.2, 100.9)	9.2 (8.4, 10.1) *	1 (0.03%)
FT3, pmol/L	4.6 (4.0, 5.3)	4.9 (4.3, 5.4)	4.1 (3.5, 4.7) *	4.7 (4.0, 5.2) *	4.7 (3.9, 5.3)	5.4 (4.9, 5.8)	1 (0.03%)
FT4, pmol/L	16.1 (14.1, 18.3)	15.4 (13.9, 17.0)	16.3 (14.3, 18.6) *	14.3 (12.9, 16.5) *	15.9 (14.8, 16.8)	20.4 (19.6, 21.6) *	0
TSH, uIU/mL	1.7 (1.1, 2.8)	1.8 (1.3, 2.7)	1.4 (0.8, 2.2) *	6.6 (5.5, 8.7) *	0.2 (0.1, 0.3) *	1.6 (1.0, 2.5) *	0
WBC, 10^9^/L	5.5 (4.3, 7.1)	5.5 (4.3, 6.9)	5.4 (4.3, 7.4)	5.7 (4.4, 7.3)	4.9 (4.0, 6.2)	5.3 (4.3, 6.9)	14 (0.4%)
Neutrophils, 10^9^/L	3.3 (2.3, 4.6)	3.2 (2.2, 4.3)	3.4 (2.5, 5.3) *	3.2 (2.3, 4.7)	3.4 (2.5, 4.4)	3.1 (2.2, 4.3)	14 (0.4%)
Lymphocyte, 10^9^/L	1.4 (1.0, 1.9)	1.6 (1.2, 2.0)	1.2 (0.8, 1.6) *	1.4 (1.1, 2.0)	0.9 (0.6, 1.3) *	1.5 (1.1, 1.9)	15 (0.5%)
N/ly	2.2 (1.5, 3.7)	2.0 (1.4, 3.0)	2.9 (1.8, 5.3) *	2.0 (1.5, 3.1)	3.1 (2.2, 6.1) *	2.0 (1.4, 3.2)	14 (0.4%)
Hemoglobin, g/L	131 (118, 146)	134.5 (123, 148)	126 (111, 142) *	128 (114, 142) *	126 (114, 136) *	135 (123, 149)	14 (0.4%)
Platelet, 10^9^/L	200 (160, 249)	203 (164, 250)	193 (150, 245) *	199 (167, 253)	195 (170, 249.5)	211 (174, 256) *	14 (0.4%)
D-Dimer, ug/ml	0.33 (0.21, 0.78)	0.26 (0.19, 0.55)	0.53 (0.25, 1.27) *	0.45 (0.23, 1.02) *	0.34 (0.22, 0.61) *	0.33 (0.20, 0.72) *	45 (1.4%)
COVID-19 Clinical outcomes							
Severe, n (%)	244 (7.7%)	40 (2.8%)	172 (14.9%) *	21 (10.0%) *	1 (3.2%)	10 (2.9%)	0
Oxygen therapy, n (%)							0
No	2534 (80.2%)	1245 (87.8%)	801 (69.4%) *	164 (77.7%) *	28 (90.3%)	296 (85.3%)	
Oxygen inhalation	443 (14.0%)	154 (10.9%)	208 (18.0%) *	34 (16.1%)	2 (6.5%)	45 (13.0%)	
Non-invasive assisted breathing	113 (3.6%)	11 (0.8%)	86 (7.5%) *	10 (4.7%) *	1 (3.2%)	5 (1.4%)	
Invasive mechanical ventilation	71 (2.2%)	8 (0.6%)	59 (5.1%) *	3 (1.4%)	0	1 (0.3%)	
Hemodialysis treatment, n (%)	68 (2.2%)	10 (0.3%)	52 (1.6%) *	4 (0.1%)	0	2 (0.1%)	0
Time to negative^2^, days	12 (9, 16)	12 (9, 15)	12 (9, 16)	12 (8,17)	11 (4, 15)	12 (9,16)	19 (0.6%)
Hospital stays, days	10 (5, 13)	9 (5, 12)	11 (7, 15) *	9 (5, 13)	11 (4, 15)	10 (6,14) *	6 (0.2%)

^1^ Waiting time defined as days from SARS-CoV-2 infection confirmed to admission, ^2^ Time to negative defined as days from SARS-CoV-2 infection to SARS-CoV-2 RNA CT<35 at least twice in consecutive days; * Compared with the normal group, P<0.05. NTIS non-thyroidal illness syndrome, HbA1C glycated hemoglobin, ALT alanine transaminase, AST aspartate transaminase, LDH lactate dehydrogenase, ALB albumin, eGFR estimated glomerular filtration rate, ULN upper normal limit, K serum potassium, Na serum sodium, Ca serum calcium, TT3 total triiodothyronine, TT4 total thyroxine, FT3 free triiodothyronine, FT4 free thyroxine, TSH thyrotropin, WBC white blood cell, N/ly, neutrophil-to-lymphocyte ratio.

The incidence of severe COVID-19 was as high as 14.9% among the NTIS group, followed by hypothyroidism, thyrotoxicosis, and the unclassified group, which was 10.0%, 3.2%, and 2.9%, respectively, compared with 2.8% in normal thyroid function group, see **Table 1**. Regarding oxygen therapy, 30.6% of the NTIS group were prescribed oxygen therapy during hospital stay, which was as low as 12.2% in the normal thyroid function group. The oxygen therapy comprised oxygen inhalation, non-invasive mechanical ventilation, and invasive mechanical ventilation, which were 18.0%, 7.5%, and 5.1% in the NTIS group. Compared with the normal group, NTIS patients had more extended hospital stays (median 11 vs. 9 days).

### Association of thyroid function patterns and severe COVID-19

**Figure 2:**
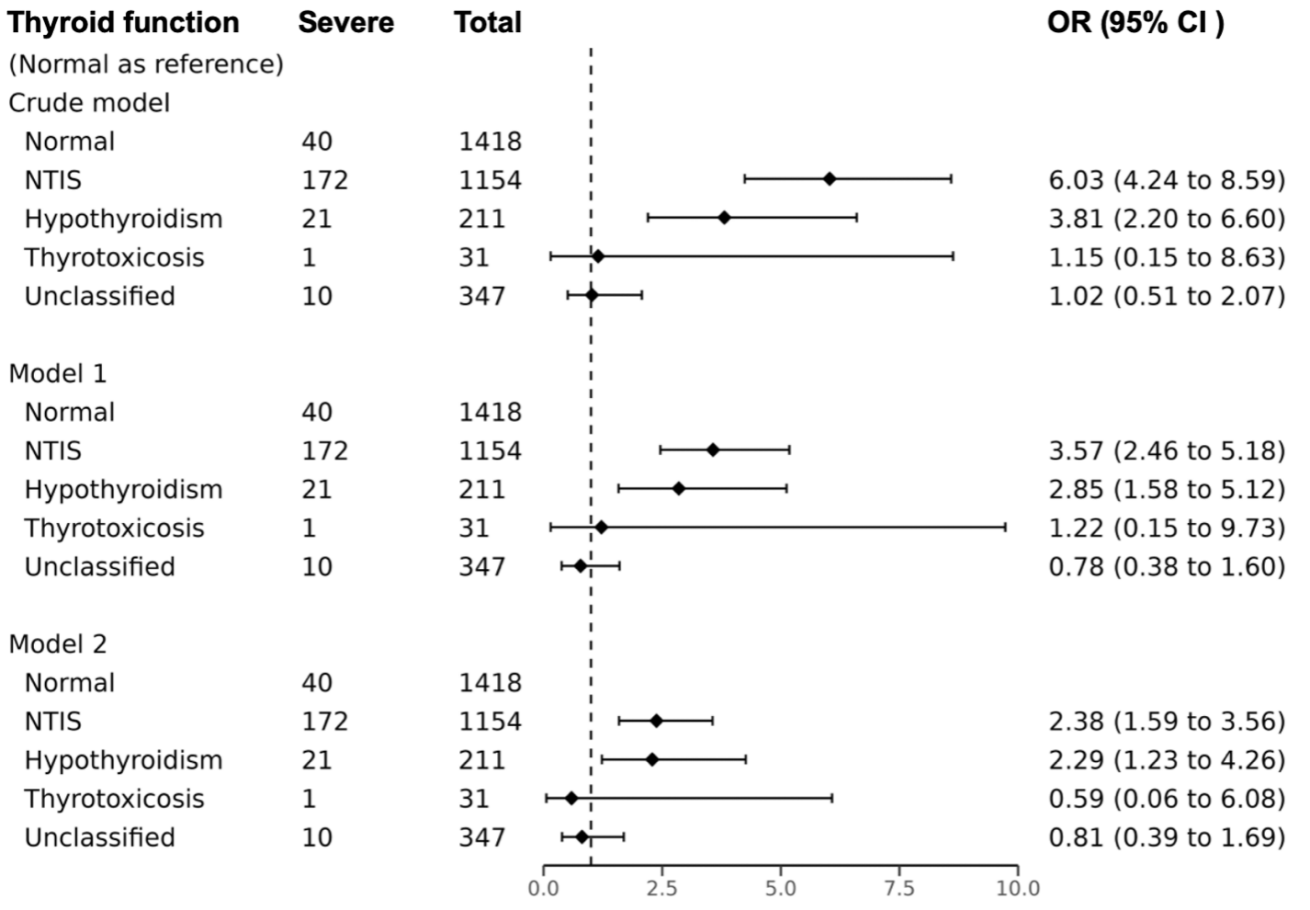
Association between thyroid function patterns and severe COVID-19. Model 1 multivariate logistic regression model adjusted age, sex, diabetes, and vaccination; Model 2 multivariate logistic regression model adjusted age, sex, diabetes, vaccination, AST≥3ULN, eGFR<30 ml/min/1.73m^2^, Ca, WBC, N/ly, D-Dimer. OR odds ratio, CI confidence interval, NTIS non-thyroidal illness syndrome. Missing data were filled using the K-nearest neighbors (KNN) method with a value of K=10.

The odds of severe COVID-19 were significantly higher in NTIS and hypothyroid patients, as indicated by unadjusted ORs of 6.03 (95% CI, 4.24, 8.59, P<0.001) and 3.81 (95% CI, 2.20, 6.60, P<0.001) respectively, compared to normal thyroid function patients, **Figure 2**. In model 1, after adjusting for age, sex, vaccination status, and diabetes, the ORs remained high at 3.57 (95% CI, 2.46, 5.18; P<0.001) for NTIS, and at 2.85 (95% CI, 1.58, 5.12; P<0.001) for hypothyroidism. In model 2, additional adjustment for clinical characteristics at admission resulted in ORs of 2.38 (95% CI, 1.59, 3.56; P<0.001) for NTIS and 2.29 (95% CI, 1.23, 4.26; P<0.001) for hypothyroidism.

### Association of TT3 levels and severe COVID-19 clinical outcomes

**Figure 3:**
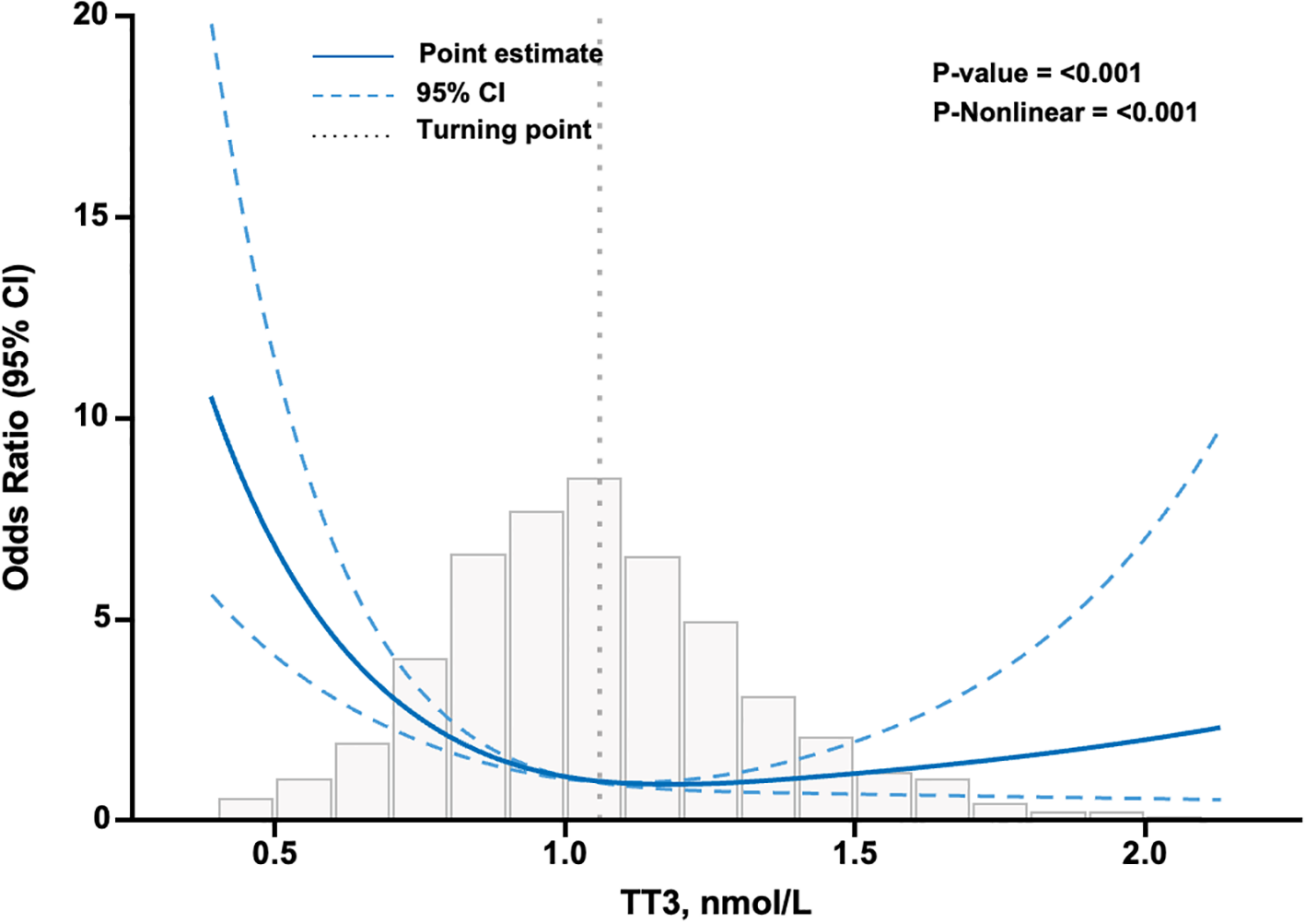
Association between TT3 level and severe COVID-19 in patients infected with SARS-CoV-2 using restricted cubic splines. The model has 3 knots at 10th, 50th, and 90th percentiles. The Y-axis represents the OR of severe COVID-19 for any value of TT3 compared to individuals with a TT3 level of 1.06 nmol/L. The logistic regression was adjusted for age, sex, diabetes, vaccination, AST≥3ULN, eGFR<30 ml/min/1.73m2, Ca, WBC, N/ly, and D-Dimer. Missing data were filled using the K-nearest neighbors (KNN) method with a value of K=10. N=3161. CI confidence interval, TT3 total triiodothyronine.

The RCS analysis showed that the curve in **Figure 3** was an L-shape and suggested a non-linear association. The inflection point of the RCS curve was identified at TT3 = 1.06 mmol/L, representing a turning point in the relationship between the TT3 level and severe COVID-19. Both NTIS and hypothyroid patients had TT3 levels below the turning point of 1.06 nmol/L. In the NTIS and hypothyroidism subgroups, after adjustment for clinical characteristics as in Model 2, the odds of severe COVID-19 decreased significantly with each increment of 0.1 nmol/L of TT3 level, 0.73 (95% CI, 0.64, 0.82; P<0.001).

### Association of TT3 levels and inflammatory factors and lymphocyte subset

Significant negative correlations were found between the TT3 levels and T lymphocyte subsets in SARS-CoV-2 infected patients, as shown in **Supplementary Figure 1A-C**. Furthermore, TT3 levels were negatively correlated with inflammatory cytokines, including hs-CRP, SAA, and IL-6, especially among the NTIS and hypothyroidism subgroups, as shown in **Supplementary Figure 1D-F** and **Supplementary Table 2**.

## Discussion

The prevalence of NTIS was observed in 36.5% of patients upon admission in this study. NTIS has been commonly reported among individuals with SARS-CoV-2 infection (7, 9, 10), with rates ranging from 1.7-66.3%, mainly depending on study cohorts and disease severity (19). This study confirmed that NTIS was associated with severe clinical outcomes, with NTIS patients having 2.4 times the odds of developing severe COVID-19 compared to the normal thyroid group after adjusting for multiple relevant clinical characteristics. Additionally, NTIS patients had more extended hospital stays and a higher need for oxygen therapy compared to the normal thyroid function group. NTIS can be recognized as an early indicator of severe COVID-19, supporting the conclusions of previous research (5, 7, 9). Previous research has demonstrated that NTIS was linked to a 3.2-3.5 times greater risk of severe COVID-19 (7, 9). However, there are discrepancies in the literature regarding the study populations, the definition of NTIS, and the reference group used. In this study, we included 3161 SARS-CoV-2 infected patients with varying severity of COVID-19. The definition of NTIS used in this study was based on low TT3 levels, while some studies used a low FT3 level and non-NTIS patient as a reference group.

In this study, hypothyroidism only accounts for 6.7% of the study population, we still observed an elevated risk of severe COVID-19 in hypothyroid patients, that hypothyroid patients were associated with 2.3-fold higher odds of developing severe COVID-19 compared to the normal thyroid group. Similar results were also noticed in a retrospective study, in which 7.23% of patients had subclinical hypothyroidism, and the hazard ratio (HR) of severe COVID-19 was 4.04 compared to those with normal thyroid function (20). In certain circumstances, distinguishing between hypothyroid patients and those with NTIS can be challenging. Since NTIS is a complex condition, it demonstrates heterogeneity in the progressive stages of the disease. Notably, during the recovery phase of chronic illnesses, it may present as elevated TSH levels (21). We speculate there exists an overlap between NTIS and hypothyroidism in this study. At present, the research on the association between hypothyroidism and severe COVID-19 is limited. Additional studies are required to understand and verify this association comprehensively.

Interestingly, this elevated risk of severe COVID-19 in hypothyroid patients was found to be comparable to that of NTIS patients in model 2. Since low plasma TT3 levels were the most consistent and significant alteration in NTIS and hypothyroid patients, further investigation was conducted to explore the correlation between TT3 levels and severe COVID-19. Among NTIS and hypothyroid patients, higher levels of TT3 were associated with lower risks of severe COVID-19 (OR 0.73, 95% CI 0.64-0.82, every 0.1nmol/L increment in TT3 level). TT3 levels have been observed to decrease shortly after surgery (22) and persist throughout the disease in both acute and chronically ill patients (23). This decrease in TT3 level becomes more pronounced as the disease worsens (6, 24, 25). Other studies have also reported the correlation between TT3 levels and the severity of COVID-19. At the early start of the COVID-19 epidemic, one retrospective cohort study including 50 patients concluded that the degree of TT3 decrease correlated with the severity of COVID-19 (26). Another study carried out among 119 SARS-CoV-2 infected patients presented that the patients in the lowest TT3 tertile (HR, 5.07) and the middle tertiles (HR, 2.05) had higher risks of death compared with those in the highest TT3 tertile (8). In addition to TT3, decreased FT3 levels have been reported to be an independent factor for poor clinical outcomes and death in mild and critical COVID-19 patients (7, 9, 10, 27). According to our knowledge, this is currently the only study that quantitatively describes the correlation between the extent of TT3 reduction and the increased risk of severe COVID-19.

This study observed a significant negative correlation between TT3 levels and proinflammatory biomarkers such as hs-CRP, SAA, and IL-6, especially in the NTIS and hypothyroidism subgroups, see **Supplementary Figure 1** and **Supplementary Table 2**. This finding aligns with previous studies that have also reported a negative correlation between T3 levels and hs-CRP (8), SAA (28), and IL-6 (7) levels. We speculate that pro-inflammatory factors may catalyze changes in thyroid function. Although the exact pathophysiological mechanisms linking thyroid function and SARS-CoV-2 infection are not fully understood, it is plausible that COVID-19 infection can induce inflammation and trigger immune responses (29), impacting various organs, including the thyroid-pituitary-hypothalamic axis. Previous research has shown that proinflammatory cytokines play a significant role in developing NTIS in severe patients (30). There is decreased pulsatile secretion of TSH and impaired TSH response to low levels of circulating T3 and T4 at the pituitary level (31, 32). Additionally, in peripheral tissues, pro-inflammatory cytokines have been found to suppress deiodinase 1 and increase deiodinase 3, leading to a sudden decrease in T3 levels and an increase in reverse T3 (rT3) in circulation (33). Considering the potential impact of SARS-CoV-2 infection, it is reasonable to suggest that proinflammatory factors may also be the driving force behind changes in thyroid function.

This study also revealed a positive correlation between TT3 levels and lymphocyte subset counts, particularly with the CD3+ subset (Spearman’s rho 0.331, P<0.001). Other studies have reported similar findings, showing that TT3 levels, rather than TSH or other thyroid hormones, are associated with lymphocyte counts in patients with bacterial sepsis (34) and that FT3 levels correlate with lymphocyte counts in COVID-19 patients (35, 36). Previous research has suggested that thyroid function plays a role in regulating cell-mediated immunity and lymphocyte proliferation. In healthy individuals, TT3 and TT4 levels were positively linked to various lymphocyte subgroups, particularly memory T cells, natural killer T cells, and CD3+/CD4+/CD45RO+ memory T helper cells (37). People with hypothyroidism presented a decrease in lymphocyte function, which could be restored after exogenous hormone administration (38). Therefore, TT3 levels may serve as a reliable indicator for assessing disease severity and predicting clinical outcomes in COVID-19. Our subgroup analysis observed weaker correlations among patients with normal thyroid function. However, the correlations were more significant in the hypothyroidism and NTIS subgroups, as shown in **Supplementary Table 2**.

This study focused on the TT3 level instead of the FT3 level. Since it is widely accepted that a decrease in TT3 level is positively correlated with the severity of the disease. Additionally, the reduction in TT3 amplitude is generally more significant than that of FT3 (23). Furthermore, our study cohort found that decreased TT3 levels were more common than decreased FT3 levels (39.1% vs. 1.5%). All 46 patients with low FT3 levels in our study also had low TT3 levels. When exploring the association between T3 and severe COVID-19, we identified a turning point for TT3 at 1.06 nmol/L, which is close to the lower limit of the reference range for TT3 based on immunoassays. Considering the convenience in clinical practice, we ultimately decided to use low TT3 levels in defining NTIS.

This study has several limitations that should be considered. Firstly, it was conducted at a single center, which may limit the generalizability of the findings. However, the study population included over three thousand individuals with varying degrees of SARS-CoV-2 infection, which partially enhances the generalizability of the results. Secondly, there was a lack of medical history regarding preexisting thyroid disease and potential drug confounders that could interfere with the accurate detection of thyroid function. Thirdly, it is essential to note that thyroid function was tested within 24 hours of admission before any subsequent treatment was administered during hospital stays. Fourth, we relied solely on medical history, medication use, or HbA1c levels to confirm the presence of diabetes, which may have excluded certain individuals. Lastly, the absence of follow-up to track changes in thyroid function is another limitation of this study. Future research should incorporate follow-up assessments to provide a more comprehensive understanding of the long-term effects on thyroid function in patients with SARS-CoV-2 infection.

## Conclusion

Patients with thyroid hormone profiles of NTIS or hypothyroidism on admission were associated with increased risks of developing severe COVID-19 during hospital stays. The decreased level of TT3 correlated with the increased risk of severe COVID-19 in patients with NTIS or hypothyroidism.

## Data Availability

The original contributions presented in the study are included in the article/**Supplementary Material**, further inquiries can be directed to the corresponding author/s.
