# Supplementary material for: Association between thyroid function and prognosis of severe COVID-19 among patients with SARS-CoV-2 infection: a retrospective cohort study in China
Source: Front Endocrinol (Lausanne). 2024 Sep 24;15:1361479. doi: 10.3389/fendo.2024.1361479 (PMC11458384; doi:10.3389/fendo.2024.1361479)
Supplement: Supplementary file 1 [file DataSheet1.docx]

Supplementary Material

**Title:** Association between thyroid function and prognosis of severe COVID-19 among patients with SARS-CoV-2 infection

**Yaling Yang^1^,** **Qian Lifang^2^, Chenwei Wu^1^, Xinyue Xu^1^, Duoduo Qu^1^, Lihua Zhou^1^, Jia liu^1^, Qin Zhu^1^, Chunhong Wang^1^,** **Xiaolong Zhao^1,^ ***

1 Department of Endocrinology, Shanghai Public Health Clinical Center, China.

2 Department of Medicine, Shanghai Qingpu District Zhujiajiao People's Hospital, China.

*** Correspondence**: Xiaolong Zhao: zhaoxiaolong@fudan.edu.cn

**Supplementary Table 1.**  Definition of thyroid function patterns among the 3161 patients with SARS-CoV-2 infection

| Thyroid function patterns | Subgroup | Patient,  n | Percentage, % |
| --- | --- | --- | --- |
| Normal | TT3 normal + FT4 normal + TSH normal | 1418 | 44.9 |
| NTIS | TT3↓+TSH normal | 1072 | 33.9 |
|  | TT3↓+TSH↓ | 82 | 2.59 |
| Hypothyroidism | Overt: FT4↓+TSH↑ | 5 | 0.16 |
|  | Subclinical: FT4 normal +TSH↑ | 206 | 6.52 |
| Thyrotoxicosis | Overt: TT3↑+ FT4↑+ TSH↓ | 2 | 0.06 |
|  | Mild: TT3↑+ FT4 normal +TSH↓ | 2 | 0.06 |
|  | Subclinical: TT3 normal + FT4 normal + TSH↓ | 27 | 0.85 |
| Unclassified | TT3 normal + FT4↑+ TSH↓ | 16 | 0.51 |
|  | TT3 normal + FT4↓+ TSH normal | 2 | 0.06 |
|  | TT3↑+ FT4 normal+ TSH normal | 2 | 0.06 |
|  | TT3 normal + FT4↑+ TSH normal | 309 | 9.78 |
|  | TT3↓+ FT4↑+ TSH↑ | 6 | 0.19 |
|  | TT3 normal + FT4↑+ TSH↑ | 12 | 0.38 |

NTIS non-thyroidal illness syndrome, TT3 total triiodothyronine, FT4 free thyroxine, TSH thyrotropin.

**Supplementary Table 2.** Correlation between TT3 levels and lymphocyte subsets and inflammatory factor.

| **Subgroup** | **Hs-CRP** | | **SAA** | | **IL-6** | | **CD3+** | | **CD4+** | | **CD8+** | |
| --- | --- | --- | --- | --- | --- | --- | --- | --- | --- | --- | --- | --- |
|  | rho | N | rho | N | rho | N | rho | N | rho | N | rho | N |
| Normal | -0.052 | 884 | -0.097^**^ | 1095 | -0.078^**^ | 1339 | 0.137^**^ | 1386 | 0.134^**^ | 1386 | 0.100^**^ | 1386 |
| NTIS | -0.382^**^ | 872 | -0.270^**^ | 897 | -0.290^**^ | 1076 | 0.260^**^ | 1133 | 0.287^**^ | 1133 | 0.199^**^ | 1133 |
| Hypothyroidism | -0.413^**^ | 149 | -0.334^**^ | 179 | -0.330^**^ | 199 | 0.297^**^ | 206 | 0.307^**^ | 206 | 0.237^**^ | 206 |
| Thyrotoxicosis | -0.39 | 22 | -0.140 | 24 | -0.195 | 27 | 0.554^**^ | 27 | 0.461^*^ | 27 | 0.401^*^ | 27 |
| ﻿Unclassified | -0.012 | 232 | -0.075 | 277 | -0.066 | 330 | 0.076 | 337 | 0.039 | 337 | 0.092 | 337 |

**P<0.01; *P<0.05; rho, Spearman‘s rho; Hs-CRP, high sensitivity C-reactive protein; SAA, serum amyloid A; IL-6, interleukin-6. TT3, total triiodothyronine.

**Supplementary Figure 1. Scatterplots of TT3 levels and inflammatory factors and lymphocyte subsets**


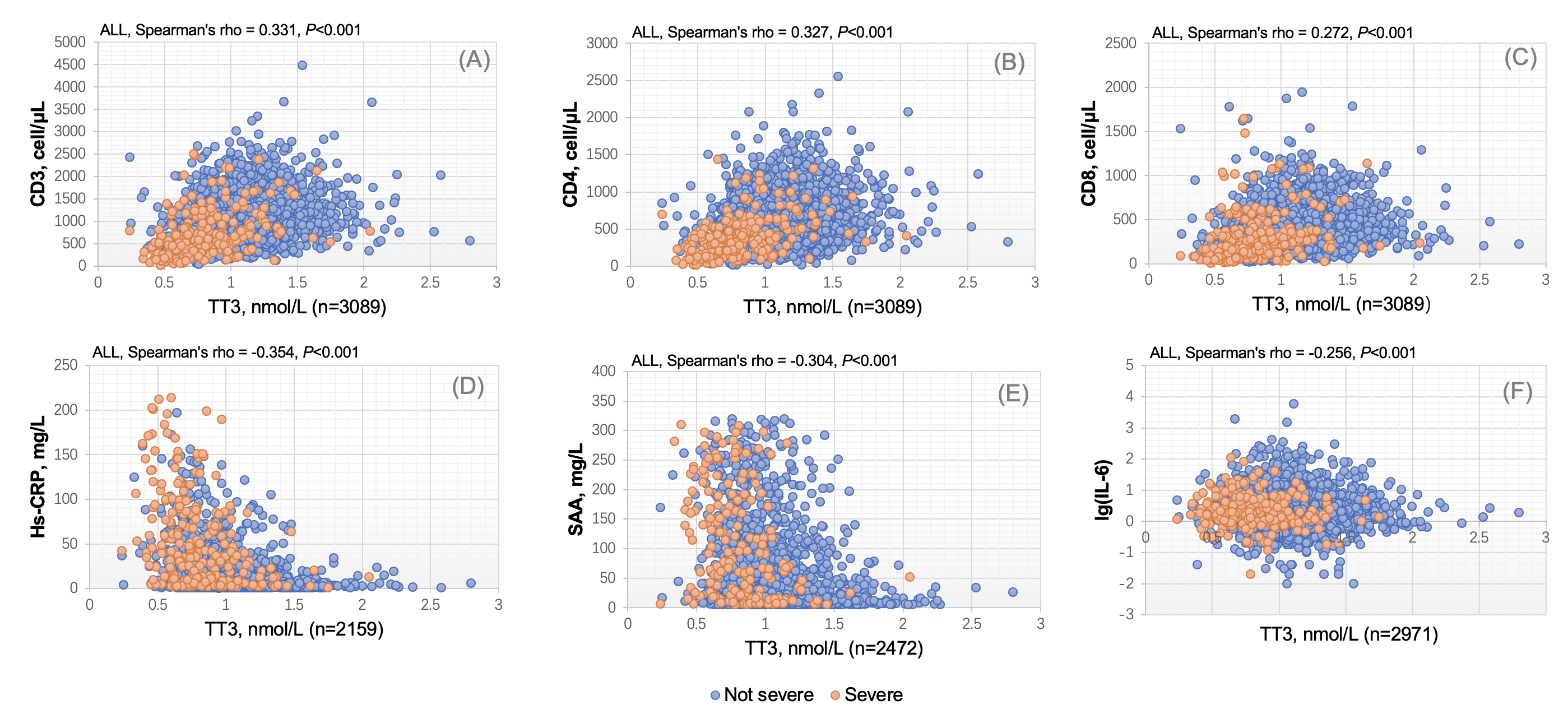


Hs-CRP, high sensitivity C-reactive protein; SAA, serum amyloid A; IL-6, interleukin-6. TT3, total triiodothyronine.
